# (Mis)perception of bias in print media: How depth of content evaluation affects the perception of hostile bias in an objective news report

**DOI:** 10.1371/journal.pone.0251355

**Published:** 2021-05-26

**Authors:** Yana Litovsky

**Affiliations:** Department of Social and Decision Sciences, Carnegie Mellon University, Pittsburgh, Pennsylvania, United States of America; Arizona State University, UNITED STATES

## Abstract

The hostile media effect describes the tendency for partisans to evaluate media content as relatively biased against their positions. The present study investigates what specific contextual elements of a news report contribute to this effect and how it may be mitigated by the depth of content evaluation. A online study of 102 participants revealed that less bias is perceived in a newspaper article when evaluating specific aspects of the article with the text available for reference than when evaluating the overall bias without referring to the text. Moreover, being asked to consider overall article bias increased subsequent ratings of bias in the discrete elements of the text. These results suggest that the perception of media bias may be counteracted by encouraging deep, evidence-based considerations of where the alleged bias might lie, but only if this happens before the reader has the chance to form an opinion based on a cursory assessment.

## Introduction

So many of the social, cultural and political issues which impact our lives and shape our perception of society are defined for us by the media coverage they receive. Where anecdotal experience is scant and personal expertise missing, the information derived from newspapers, magazines and television news programs may constitute our main source of insight into a given issue and—as mediated by the effect of prior attitudes and persuasive advertising—become incorporated into our unique perspective on the facts.

While media provide the building blocks of this attitude formation, our perception of reality is by no means a direct function of the news we are exposed to. Quite simply, we don’t always believe what we read or see. Whether it is a mistrust of the newsmakers’ accuracy or agenda, accusations of media bias are rampant. A 2005 poll showed that only 50% of Americans trusted the media to report news truthfully and fairly, compared to 70% in 1972 [1], and the public perception of the media as having a liberal bias in particular has been widely documented [2]. The perception of bias has hardly improved in recent years—a 2020 poll found that 49% of adults saw a great deal of political bias in their news coverage [3].

If we define bias as any unintentional and undisclosed subjective influence on the content of news, then yes, it is certainly unavoidable, since that content is selected by fallible humans, from infinite possible story options and permutations of fact. But if bias is defined, more narrowly and realistically, as the failure to apply some normative standard to a particular issue in violation of basic journalistic standards of neutral, objective reporting, then there is ample evidence that the contemporary American media landscape is far less slanted than we may perceive. Media bias can stem from individual news workers, organizational routines (the standardized rules used by those workers) and larger organization goals to lure an audience with spin or sway it with ideology [4]. And while evidence of this bias has been reported by a number of studies [5–7]—these empirical accusations of bias are strongly counterweighted by many other reports that it is minimal and not systemic. A meta-analysis, published in 2000, of 59 quantitative studies concluded that negligible bias existed in news coverage of presidential election campaigns since 1948 [8]. While accusations of bias in the news, fueled by an extremely polarized political climate, seem to have increased in recent years, a paper published in 2020 again found no evidence of liberal bias in political news coverage [9].

Based on the evidence, both empirical and anecdotal, it can be argued that overall, we perceive more bias in the media than there actually exits. More specifically, it has been found that *partisans* on *controversial* issues are more likely to perceive coverage of those issues as biased, and, more specifically, to perceive that coverage as biased against their own interests. Known as the hostile media effect (HME), this tendency was first described by Vallone, Ross and Lepper in their seminal 1985 study [10]. Participants on opposite ideological fronts of the Israeli/Arab conflict rated identical samples of major network television coverage of the 1982 Beirut massacre as having been slanted in favor of the other side, likely to sway a neutral person toward that side and produced by people with opinions that are the opposite of their own.

### Scope of hostile media effect research

Building on Vallone, Ross and Lepper’s research paradigm, a number of follow-up studies have validated the existence of this phenomenon across different issues, such as the 1997 UPS Strike [11], use of primates in lab research [12], genetically modified food [13] and presidential performance [14]. In each case, the studied media communication, whether print or video, was either pre-screened for fairness and presented in an experimental context [10,15], or, the lack of significant media bias was presupposed and participants were simply questioned about their perception of bias in the news they already consumed [16].

Numerous moderating factors—including the level of issue controversy, the perceived source and reach of the media communication, as well as some partisan traits and demographics—have also been investigated. The emerging picture from the literature is as follows: the hostile media effect is strongest when the issue is divisive, the partisan viewer or reader is highly involved, the communication is a product of mass media and its audience is perceived to be vast and vulnerable. A controversial topic is most likely to show evidence of HME only inasmuch as it increases the likelihood of inspiring highly ego-involved partisans on both sides. A sufficient level of ego-involvement (the degree to which a participant identifies with an ideology) is required to demonstrate this effect and the degree of such partisanship is positively correlated to the degree of perceived hostile bias [15,17].

In addition to the partisanship of the media consumer, the communication must be seen as a far-reaching product of the media in order to trigger the perception of hostile bias. There is evidence that the HME can be erased, or even reversed, if identical information is presented in a non-media context [13]. While participants perceived a hostile slant in an article presented as news coverage, they found neutral and even favorable bias when the same communication was presented as a college student’s composition. A follow-up study was conducted to identify which distinct aspect of the non-media context is responsible for HME-suppression. This was done by untangling the influence of the perceived audience from the perceived characteristics of the author [17] and it was found that both low-audience and student-authorship conditions independently eliminated the reporting of hostile bias. Conversely, suggesting that either the author was a journalist or the article was intended for national circulation produced strong evidence of the HME.

Tying these components together is the hypothesis that a partisan’s fear of the media’s ability to persuade a neutral audience in the “wrong” direction on a vital topic is one of the main considerations driving their misperception of hostile bias. A recent poll shows that Americans are much more concerned about bias in the news other people are getting (69%) than about their own news being biased (29%) [3].This may be due, in part, to illusory superiority, such as the bias blind spot [18], which describes our tendency to see others as more susceptible to bias than ourselves or the third-person effect, in which partisans assume others to be disproportionately affected by arguments or facts supporting the wrong side of an issue [19].

Highly involved partisans are especially prone to these biases since they tend to be more sensitive about the opinions of others, perceive others as more vulnerable and less informed, and are more likely to consider the influence of the news on its audience [11]. So by removing concern for this “vulnerable” audience, the partisan reading a non-media communication should display confirmation bias and summarily accept attitude-congruent information while carefully evaluating and often dismissing unfavorable content as unreliable [for a review of confirmation bias, see 20]. While it may seem surprising that HME can so drastically reverse the ubiquitous information processing approach of skewing evidence toward (not against) our attitudes, both tendencies are similarly based on our proclivity to cling to our beliefs, either by tinting contradictory evidence as favorable (in the non-media scenario) or by dismissing it as biased.

But what is it about the nature of information presented in a media context that converts a person’s more natural tendency for bias assimilation into bias vigilance? One of the information processing mechanisms proposed to explain a partisan’s attribution of hostile bias to verifiably balanced coverage is *selective recall*. Highly ego-involved parties on a given issue may be attending to or recalling different facts when analyzing the same article or news footage, leading pro-Palestinian partisans, for example, to recall more reports of Palestinian transgressions than Israeli ones in a given communication [10]. Though partisans are more likely to attend to attitude-*congruent* information outside of the media context, perhaps the *incongruent* information in the news derives its salience by violating the partisans’ highly guarded conceptualization of the truth, and, in turn, activating a more elaborate thinking process necessary for internal counterargument [Higgins & Bargh, 1987; as cited in 15]. There is evidence that information inconsistent with one’s preferences is more deeply and systematically processed [21]. In that case, unfavorable information in an article might be more thoroughly encoded and more readily remembered, perhaps explaining why two partisans on opposite ends of an issue spectrum may recall the landscape of a report very differently. Subsequent researchers have tested the validity of the *selective recall* explanation using another, arguably less idiosyncratically polarizing issue–the use of genetically modified foods–and found that the HME was better explained by selective *categorization* of facts as pro- versus counter-attitudinal, rather than by selective recall [13]. However, given the scarcity of studies explicitly exploring the mechanism behind the HME, the exact cognitive processes responsible for this phenomenon remain uncertain.

### The present research

While we have a firm understanding of *when* hostile bias is perceived and a tenuous understanding of *how* the information may be processed, there is another avenue of research that has been largely overlooked. We do not know what elements of an article a reader is attending to when gauging a hostile slant. And, perhaps more importantly, we do not know whether provoking deep and deliberate consideration of these elements increases or decreases the perception of bias. Would readers who might allege bias after reading an article be less likely to do so if they were induced to consider which, if any, of the article’s constituent parts were unduly slanted?

To fill this gap in the literature, the present study will assess how various distinct features of an article are interpreted by the reader when considering the presence of bias in a news report and aim to determine if and how these specific features relate to a general assessment of bias in the same report. The goal is to understand how our judgments about the fairness or objectivity of an article’s language or choice of evidence, for example, compare to a big-picture judgment about the overall degree of article objectivity. Assuming that an article is in fact objective and that, as the HME literature suggests, partisans are likely to invent or exaggerate the presence of hostile bias, will it be more difficult to activate the confirmatory information search or information avoidance needed to convince ourselves that such bias exists when focusing on specific elements of a short news communication rather than evaluating the article as a whole?

Although there is some evidence that careful deliberation and conscious thought might, sometimes, result in sub-optimal weighting of individual components of the issue under consideration and a poorer ultimate decision [22,23], these findings should not apply to the type of “deliberation” activated by the analysis of the article on a micro-level, as defined here. The reader will not simply be asked to consider the article more deeply but to consider specific, pre-determined aspects of the article with the text available for reference. If indeed the HME is the product of various biases (such as the better-than-average effect) and biased cognitive strategies (such as selective recall or selective categorization), being forced to consider and identify the specific presence of impartiality in an article should attenuate the effect. For one, careful consideration of the text should activate the use of System 2 reasoning to override the partisan’s System 1 tendency to rely on the heuristics which contribute to the perception of media communications as hostile [for review see 24]. Secondly, if selective recall is, in fact, the dominant cognitive strategy responsible for HME, then being explicitly presented with the components of the article to consider, while the article is available for review, should inhibit selective recall of information and, in turn, the strength of the HME. Finally, it seems intuitive perhaps that if an article truly does not have an obvious slant or agenda, then one would be less liable to perceive such a slant after being directed toward specific, concrete and readily comprehensible aspects of the story.

The first research question is: will *less* hostile bias be perceived in the assessment of individual elements of a news report (what I will refer to as “micro-level” bias) than in the assessment of article balance on the whole (“macro-level” bias)? I predict that the assessment of micro-level bias will be lower than the assessment of macro-level bias because the misperception of bias will be strongest when heuristics are not challenged by deep, evaluative thought about the content of the article. And conversely, when an article is evaluated with attention to the micro elements of content, the partisan should be better able to gauge the article as an objective source of information.

Beyond comparing micro- and macro-level bias perception, I am also interested in the influence of one method of content evaluation on the other. First, I want to know how micro-level evaluation changes subsequent macro-level evaluation of the same content. If, as suggested in the first hypothesis, the misperception of hostile bias should be less likely after deeper micro-level analysis, then it may follow that being asked about an article’s overall level of bias after being asked about the level of bias in the specific individual elements of content would produce a lower, more realistic assessment of overall bias. So, the second research question is: does first evaluating the micro-level bias change the macro-level assessment of bias? I predict that less macro-level bias will be perceived if assessed *after* the evaluation of article bias on a micro-level because the deeper cognitive processing will prime considered thought and counteract the errors of judgment responsible for HME.

Finally, I will also examine how priming the reader with a macro-level evaluation impacts micro-level bias assessment. The third research question is therefore: does first evaluating the macro-level bias change the micro-level assessment of bias? In this case, the reader may now report *more* micro-level bias because s/he will have already declared the extent to which the article is generally biased and may now look for confirmatory evidence in the contextual cues. So, I predict that more micro-level bias will be perceived if assessed after the evaluation of article bias on a macro-level because the reader will look for confirmatory evidence of the bias s/he has already reported.

This study will pursue these three research questions in an effort to expand our understanding of how specific elements of a news report may protect against the inaccurate perception of hostile bias. If the misperception of hostile media bias can be attributed to shallow reading, this finding will further not only HME research but also the conversation about how journalists and educators can better safeguard against the pernicious illusion of media bias which hinders the apprehension of objective information and encourages readers, convinced that impartial news is impossible, to retreat into their own ideological camps.

## Materials and methods

### Participants

102 participants were surveyed via Amazon’s Mechanical Turk (58 men, M_age_ = 34, SD = 10.48). (Two respondents completed the survey but were eliminated from the dataset because they did not correctly answer an attention check question). Respondents received $2 for taking the 15- to 20-minute survey. Only participants who were U.S. residents and had a 95% or higher approval rating on Amazon Turk were eligible for the study.

The Carnegie Mellon University Institutional Review Board (IRB) reviewed and granted approval of this study on April 14, 2016. The study’s IRB ID number is STUDY2016_00000092: Hostile Media Effect. All participants signed an online consent form declaring that they were over 18 years old and that they agreed to participate in the study.

### Materials & procedure

Each participant was first asked to read a news article described to them as “related to recent events in the Israeli/Palestinian conflict” and as “published in 2015 in a widely-read US media outlet.” The newspaper where this article was published was not disclosed to prevent pre-existing attitudes about that publication from influencing the reader. The article text was presented as an image, so that the reader could not copy the text and search for the article online. The photograph which accompanied the article was also presented. The article was originally published in the New York Times and described a knife attack targeting Israeli citizens. This topic was chosen because the Israeli/Palestinian conflict has been used numerous times in HME research and has been shown to be an effectively controversial issue for eliciting this effect. The article was a purely descriptive news account and showed no obvious evidence of any bias against either Israelis or Palestinians (as evaluated by this author).

After reading the article, the participants were randomly assigned to one of two experimental groups. One group was asked to first answer questions assessing the bias they perceived in various contextual elements of the article, with the article available for reference. The eight micro-level bias questions asked to what extent the participant believed the article showed: 1) *language bias* (poorly chosen language, such as misleading or emotionally charged words that are inappropriate), 2) *factual bias* (stretched, exaggerated or incorrect facts about the event), 3) *omission bias* (missing relevant information about the topic which probably should have been included), 4) *addition bias* (information that was not relevant to this particular event and probably should not have been included), 5) *evaluation bias* (specific instances of inappropriate opinionated rather than objective reporting), 6) *attention bias* (inappropriate or unbalanced attention devoted to the Israeli and Palestinian sides or perspectives), 7) *headline bias* (headline was not well chosen/descriptive/objective) and 8) *image bias* (photograph was not well chosen/descriptive/objective). For each question, participants indicated the extent to which they agree with the statement that such bias does or does not exist on a continuous sliding scale from 0 to 10, with 10 (strongly agree) indicating the strongest bias (after reverse coding some of the questions). An answer of 6 or greater prompted a follow-up question which asked which side the article was biased against: Israel/Israelis, the Palestinian territories/Palestinians or neither (see Appendix in S1 File for survey questions and article).

The participants were then asked to evaluate more overall, macro-level bias, this time without access to the article, by asking about: 1) *fairness bias* (the article did not treat/present both sides fairly), 2) *influence bias* (the article would make a reader with a neutral or uncertain position on the Israeli/Palestinian conflict change his or her position), 3) *overall bias* (the reporting was not fully objective), 4) *author bias* (the journalist who wrote the story likely has a bias in regard to this issue) and 5) *publication bias* (the newspaper/website which published the story likely has an institutional bias in regard to this issue). After reverse coding one of the questions, a score of 10 indicated that the respondent strongly agreed that this bias was present in the article. And again, a score of 6 or greater prompted a follow-up question to assess which side the participant believes the article was biased against. The goal of all five questions was to measure a general impression of article objectivity from different angles. The second experimental group answered the same questions but in reverse order: macro- followed by micro-level bias questions.

Next, everyone answered several attitude questions to evaluate their level of partisanship on the issue. This was done a few different ways: Participants indicated if they were “pro-Israel,” “pro-Palestinian” or “neutral.” If they indicated an allegiance with one side, they were asked to rate the level of partisanship, from “just barely” to “completely” pro-Israel or pro-Palestinian. In case people shied away from this form of strong self-identification and too few self-declared partisans were captured, they were also asked to indicate how much each side was to blame in the ongoing conflict as a less direct proxy measure of partisanship. Participants also indicated how important the conflict was to them personally. They were then asked some factual questions about the text as a further attention measure as well as a way to gauge if thorough attention and depth of encoding affect the perception of bias. Finally, some basic demographics were collected.

## Results

### Initial analyses

Evaluations of the eight forms of micro-level bias were analyzed to determine if these “bias” measures were indeed interpreted by the participant as a lack of objectivity which favored one side over another. Of all the participants who indicated at least some bias (a score of 6 or greater out of a possible 10), most respondents answered the follow-up questions by indicating that the article was either biased against the Israelis or the Palestinians (see Table 1), with an average of 20% of the follow-up responses indicating that the participant did not in fact perceive bias against either faction. One particular outlier was *image bias*; 59% of the those who said the photograph was “not well chosen/descriptive/objective” did not attribute this flaw to bias. This may be due to the fact that these participants felt there were other reasons that the photograph was poorly chosen which had nothing to do with any possible partisan slant. Those who perceived any macro-level bias were more likely to confirm that indeed this indicated a bias against one of the two sides. Only 7% of respondents who rated the macro-level bias as 6 or greater indicated that the article did not in fact favor either of the sides. See Table 1 for distribution of perceived bias for each measure.

**Table 1:** Distribution of perceived bias.

Micro-level bias	Against Israel/Israelis	Against Palestinians	Against neither side	Total observations
omission bias	21% (8)	61% (23)	18% (7)	38
attention bias	17% (6)	74% (26)	9% (3)	35
addition bias	23% (7)	51% (16)	26% (8)	31
image bias	6% (1)	35% (6)	59% (10)	17
evaluation bias	32% (7)	64% (14)	4% (1)	22
headline bias	11% (2)	61% (11)	28% (5)	18
factual bias	25% (3)	50% (6)	25% (3)	12
language bias	6% (1)	88% (15)	6% (1)	17
**Average**	**18%** (35)	**62%** (117)	**20%** (38)	**190**
**Macro-level bias**				
author bias	31% (14)	62% (28)	7% (3)	45
overall bias	30% (13)	59% (26)	11% (5)	44
publication bias	25% (9)	67% (24)	8% (3)	36
fairness bias	28% (10)	69% (25)	3% (1)	36
**Average**	**29%** (46)	**64%** (103)	**7%** (12)	**161**
**Total average**	**23%** (81)	**63%** (220)	**14%** (50)	**351**

Side the article was biased against, as indicated by respondents who agreed that each of the 13 micro and macro-level types of bias was present. Total number of observations is indicated in paratheses.

Because all of the questions measuring perceived micro-level bias were purposefully designed without the use of the word “bias,” reporting a score of 6 or greater on these bias measures but then indicating that this did not represent a bias against either side was not necessarily indicative of a random or inattentive response. Participants may have been assessing flaws in the article (e.g., poorly chosen language) that did not necessarily favor one side over the other. To test this, the distribution of perceived bias was calculated for only those participants who performed poorly on the content quiz about the article administered at the end of the study (N = 52). If the seemingly anomalous response pattern was due to a lack of attention, then, arguably, it should be most pronounced in the group of participants who were least engaged with the article. However, this was not the case: 17% of these participants showed this response pattern for the micro-level questions and only 4% for the macro-level question (11% on average across all bias measures). Nevertheless, in order to only focus on participants who did perceive a partisan slant, for both the eight micro- and five macro-level items, these responses were removed from the dataset, so that all measures of bias corresponded to the belief that one side was favored over the other.

Among the eight micro-level elements of the news article, participants were most likely to report that the article left out some relevant information about the topic which contributed to it appearing less objective. Participants were least likely to indicate that the language, presentation of facts or headline indicated any bias. Among the macro-level elements, participants were most likely to report that the article would sway a neutral reader and that the author was likely not objective in regard to this issue. See Table 2 for each individual item mean and difference between the items.

**Table 2:** Micro and macro-level bias ratings.

Micro-level bias	Mean (SD)	Bias significantly greater than the following Paired Samples T-Test)				
omission bias	4.373 (2.41)	omission bias >				
attention bias	4.108 (2.40)		attention bias >			
addition bias	4.078 (2.39)			addition bias >		
image bias	3.735 (1.95)	t = 2.16**			image bias >	
evaluation bias	3.608 (2.34)	t = 3.67***	t = 2.09**	t = 2.16**		evaluation bias >
headline bias	3.500 (2.13)	t = 3.24***	t = 2.47**	t = 2.20**		
factual bias	3.265 (2.01)	t = 5.64***	t = 3.27**	t = 3.66***		t = 2.06*
language bias	3.020 (2.06)	t = 6.27***	t = 4.24***	t = 5.05***	t = 2.78**	t = 3.44***
**Macro-level bias**						
influence bias	4.794 (2.40)	influence bias >				
author bias	4.706 (2.52)		author bias >			
overall bias	4.382 (2.63)					
publication bias	4.363 (2.42)		t = 2.01*			
fairness bias	4.157 (2.30)	t = 2.39*	t = 2.94**			

*Note*.

* p < .05

** p < .01

*** p < .001.

All eight micro-level bias items significantly correlated at the .01 level with a Cronbach’s alpha of .86. Given the good internal consistency, a single measure of “micro bias” was computed from all eight items. Similarly, all five of the macro-level bias items were significantly correlated at the .01 level with a Cronbach’s alpha of .94, so all five were averaged to compute the “macro bias” measure.

Although the purpose of this study is not to explicitly confirm the existence of the HME, the results were first checked for evidence of this effect. When using the self-declared measure of partisanship—participants indicated if they were “pro-Israel,” “pro-Palestinian” or “neutral”—no significant relationship between partisanship and likelihood to view the article as biased was observed. Furthermore, although partisanship did very weakly correlate to perception of *hostile* bias (bias against the side the participant supports), no significant effects were found. One possible issue with using the self-declared allegiance as our measure of partisanship was that few people categorized themselves into these groups (27 declaring themselves “pro-Israel” and 10 “pro-Palestinian”). (The fact that there were more pro-Israeli than pro-Palestinian partisans in our sample is somewhat surprising given the fact that Amazon Turk workers tend to be more liberal than the general population [25]. Because partisanship was measured after participants read the article, which described attacks against Israelis, it is possible that the treatment may have increased sympathy for Israel. However, the partisanship question simply asked participants to identify themselves as pro-Israel vs. pro-Palestinian (or neutral), so it is unlikely that our treatment could affect such an unnuanced, ego-related self-categorization.) On the other hand, people were more willing to signal potential partisanship when asked to indicate how much the Israelis or Palestinians were to blame for the current conflict. On a scale of 0 (no blame at all) to 10 (entirely to blame), 10 self-declared “neutral” respondents indicated that Israel was moderately or very much to blame (response of 6 or higher) while ascribing less blame to the Palestinians. Conversely, 12 such self-declared “neutral” participants indicated that the Palestinians were more to blame. Using this continuous proxy measure of partisanship, a simple linear regression did reveal an HME-consistent result: A greater likelihood to blame Israel predicted the assessment of more overall bias against Palestinians (β = .39, *t*(38) = 2.65, *p* = .01) and more micro-level bias specifically (β = .35, *t*(34) = 2.18, *p* = .036). No significant relationship was observed between likelihood to blame Palestinians and belief that the article was biased against Israelis, although this may be due to the fact that very little bias against Israel was reported in this article.

The lack of strong evidence for the HME from these data may be attributed to a number of factors. For one, without screening for partisans or surveying more respondents, the sample did not include many partisans on the issue. Of those who did indicate an allegiance with one side or the other, when asked to rate how important the issue was to them—from 0 (least) to 10 (most important)—the average scores were relatively low (*M* = 4.18 for the 27 pro-Israel respondents, *M* = 3.6 for the 10 pro-Palestinian respondents, and *M* = 2.53 for neutral respondents). Given this limitation, I will proceed with the analyses using the entire sample, not just partisans, to more broadly focus on the impact of micro- versus macro-level bias evaluation and not restrict the analyses to hostile bias in particular.

### Main research questions

**Fig 1:**
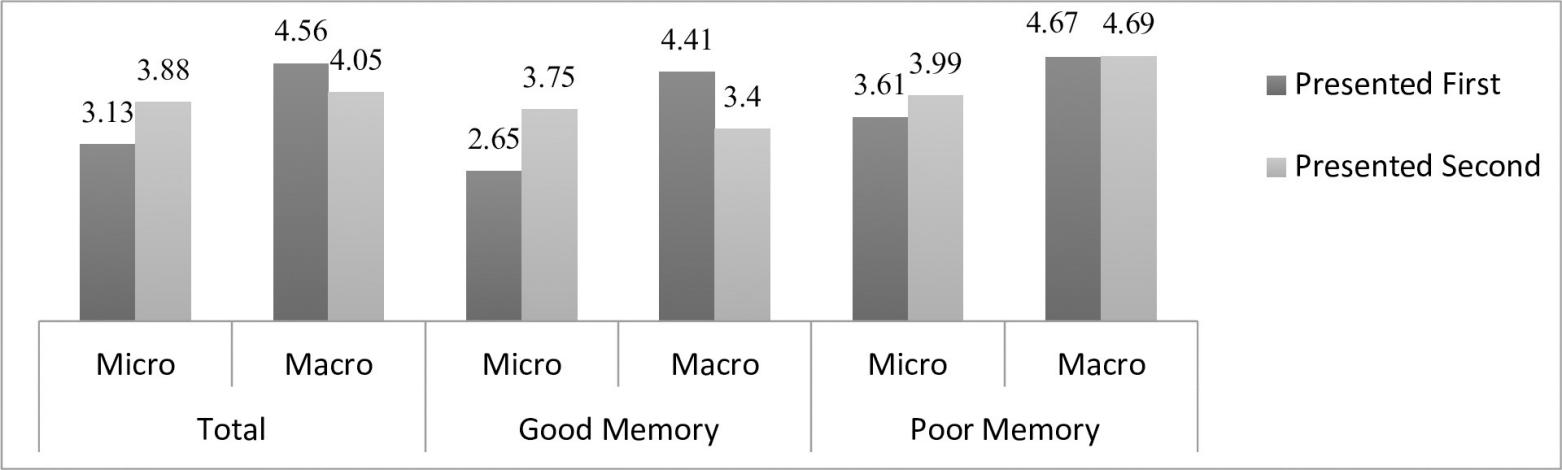
Micro and macro-level bias by order of presentation. Significant results: 1) Total first-order **macro** bias > Total first-order **micro** bias (p < .001). 2) Total **second**-order micro bias > Total **first**-order micro bias (p < .017). 3) **Poor** memory first-order micro bias > **Good** memory first-order micro bias (p = .044). 4) Good memory **second**-order micro bias > Good memory **first**-order micro bias (p = .01).

An independent t-test was conducted to determine if participants were likely to rate the article as more biased against either side when they were asked about the overall lack of objectivity as opposed to when they were asked about bias in specific aspects of the article, such as the language and choice of information. In order to avoid priming effects, only data from the *first* set of questions answered by the respondent was used—so if the micro (macro) level questions were asked after the macro (micro) level questions, these results were not included in these analyses. As predicted, the assessment of micro-level bias was significantly lower (*M* = 3.13, *SD* = 1.65) than macro-level bias (*M* = 4.56, *SD* = 2.02) (*t*(100) = 3.9, *p* < .001) (see Fig 1 for these and all subsequent results). The same relationship was observed for partisans (anyone who indicated that they were “pro-Israel” or “pro-Palestinian”) and non-partisans alike.

As a robustness check, a linear regression was conducted with the bias score from the first set of questions regressed on the type of bias that was first assessed (dummy-coded: macro-level bias = 1; micro-level bias = 0), while controlling for age, gender (dummy-coded: female = 1; male = 0); education (dummy-coded: college-educated = 1; other = 0) and political party affiliation (using one dummy-coded variable for Democrats and another for Republicans). Assessing macro- (versus micro-) level bias predicted significantly higher bias scores, (β = .34, *t*(95) = 3.64, *p* < .001), with no main effect of any of the demographic variables.

To assess the effect of asking respondents to first consider how much bias they perceive in specific aspects of the article on their subsequent evaluation of overall bias, an independent t-test was conducted to compare the aggregate macro-level measure of bias for those who were asked these questions first and those who were asked them second, after the micro-level questions. Although, as predicted, participants reported less macro-level bias if they first considered the level of bias in specific aspects of the article content (*M* = 4.05, *SD* = 2.02) than if they evaluated macro-level bias right away (*M* = 4.65, *SD* = 2.72), these results were not significant. A linear regression conducted as a robustness check revealed the same pattern of results when controlling for the demographic variables described above, with no significant main effects of any of the dependent variables.

While assessing the article’s constituent parts for explicit instances of biased reporting did not improve subsequent attitudes about the article’s overall objectivity, rating the overall bias first did increase the degree of bias perceived in the article’s individual elements. As predicted, participants reported significantly more micro-level bias if they first answered the macro-level bias questions (*M* = 3.9, *SD* = 1.45) than if they were asked these questions immediately after reading the article (*M* = 3.13, *SD* = 1.65) (*t*(100) = -2.44, *p* = .017). This effect was driven by significant changes in assessment of *headline* and *image bias*, which were the only two types of micro-level bias to be rated significantly higher after the participant considered the article’s overall level of objectivity, although the remaining six items did show a change in the same direction. A linear regression revealed that considering macro- (versus micro-) level bias first predicted significantly higher micro-level bias scores (β = .23, *t*(95) = 2.33, *p* = .022) when controlling for the demographic variables described above, with no other significant main effects.

The factual questions about the article content asked of all respondents at the very end of the survey (without the article available for reference) were coded into a binary measure to capture those respondents who answered most of them correctly and those who did not. Given the high variance in answers and large quantity of incorrect responses to the five questions, a respondent was coded as displaying “good memory” for the article if they accurately indicated where the attack took place, how many Palestinian assailants there were (this information was presented in the headline), how many Israeli victims died of their wounds (the correct answer was two but this information was not as obvious from the text so an answer of one, two or three was accepted) and if their guess for the total number of Israeli and Palestinian victims in the recent violence was within 20% of the actual value. Based on this coding scheme, 48 of the 100 respondents were categorized as having a “good” memory for the text. Independent t-tests were conducted to determine how all of the analyses above varied depending on the level of content retained by the reader. Compared to those participants with a good memory *(M* = 2.65, *SD* = 1.54), those classified as having a poor memory for the article reported significantly more micro-level bias (*M* = 3.61, *SD* = 2.65) (*t*(46) = 2.08, *p* = .044). Although participants with a poor memory also reported slightly more macro bias, this difference was not significant, which, as seen in the previous results, indicates more rigidity in the evaluation of overall objectivity regardless of attention, partisanship or order of presentation. When considering only those respondents with a good memory, being primed by overall article evaluation significantly increased the perception of micro-level bias (as seen for the total sample) (*M* = 3.75, *SD* = 1.24 vs. *M* = 2.65, *SD* = 1.54) (*t*(46) = -2.69, *p* = .01), but this effect disappeared when only those with a poor memory were considered. These results suggest that when participants read the article more attentively and/or encoded the information more deeply, they were less likely to perceive specific features of the article’s language and content as indicative of bias but, perhaps because of a lower initial rating of bias, were more likely to be swayed to see bias in the details after considering the article bias a whole.

## Discussion

This study is a broad initial attempt to define the relationship between depth of content processing and misattribution of bias in news coverage. The results support the first hypothesis that bias is most likely to be reported when a reader is assessing an article more broadly and without referring to the text. When asked to consider the level of bias in specific elements of a news report, with the text available for reference, a reader was less likely to see that article as biased against one side or the other. One possible explanation for this is that the micro-level bias evaluation prompted more consideration and deliberate reasoning than simply being asked to consider how biased the article seemed on the whole. Detailed article analysis may have counteracted the perception of bias by attenuating any cognitive tendencies which may be responsible for the perception of objective news as subjective. The HME literature, which is predominantly in the field of social psychology and communications, has not made great strides in identifying which individual heuristics or cognitive biases are directly responsible for this effect and so the present study cannot reconcile which specific cognitive tendencies may be attenuated by deliberation. But it is possible that having to access the text and having to consider where exactly the bias might lie in something as specific as a headline may interfere with our ability to use and justify motivated reasoning to see bias in the news. Further supporting this idea is the fact that the more attentive readers (those classified as having a “good memory” for the text) reported significantly less bias on the micro-level, confirming what we might already intuit: that considering an objective news report more carefully should impede our likelihood to see bias where it does not exist.

Another explanation of the significant difference between the micro- and macro-level bias reported by the participants is the interference with the process of selective recall, which has been suggested as one possible explanation for the HME. Because participants were able and encouraged to inspect the article when answering the micro-level bias questions, they were perhaps differentially *perceiving* but not differentially *recalling* the information. Two other information processing mechanisms have been proposed to explain HME: *selective categorization*, which posits that opposing partisans will perceive and recall content accurately but disagree about the valence, perhaps judging the same passage as unsympathetic to their respective sides; and *different standards*, which suggests that evenhanded coverage may be similarly attended to and understood but differently evaluated for relevance and value to the discussion [13,17]. This study may therefore provide some evidence that selective recall may be at least partially responsible for HME, given that removing the need to remember the article also lowered the perception of bias.

The third hypothesis was also supported by the results: being asked to first consider whether or not the article is generally biased, without actually going back to the text, led participants to then identify more bias in specific elements of the article, even when given the chance to refer back to the text to verify their suspicions. Although people were less likely to misperceive bias on the micro than macro level, once they considered the macro-level bias they were then more likely to confirm their initial assessment and see bias even in something as basic as the headline or the photograph. This effect was most pronounced in those participants who had read the article more carefully and better remembered the facts. This was probably because they were much less likely to *initially* identify bias on the micro-level than inattentive readers, but were still highly affected by first considering the article bias overall, accounting for the significant spike in the micro-level bias assessment from the first to the second presentation.

The failure to support the second hypothesis—that we would be less likely to see a slant in the text if first asked to consider where specifically such a slant may reside—may indicate that our inclination to see bias in an article is deeply entrenched and resistant to being challenged. Although the results were not significant, there was a tendency for respondents to report less macro bias after they were asked to consider the article in detail (especially for those with a good memory for the text), suggesting a trend which might warrant further research.

### Limitations & future direction

The main limitation of the current study is that the sample does not capture enough highly involved partisans to be able to firmly replicate the hostile media effect and, more importantly, to be able to focus the study exclusively on partisans who reported hostile bias in the article. Since highly opinionated news consumers are most likely to dismiss an objective news report as biased and rely on truly biased news coverage as their main source of information, this is the group that this research should target, in an effort to identify which nudges can reduce their tendency to see bias where it does not exist. Future studies should therefore consider how to reach more partisans, by either screening Amazon Turk workers and only surveying those with strong, partisan beliefs or by reaching out to self-identified partisan groups in certain geographic or online communities.

Another benefit of screening participants before they begin the study (as opposed to collecting this information at the end of the study) is that it would avoid the introduction of post-treatment bias [26]. However, given the evidence that answering survey questions does not change people’s reported political attitudes [27], this particular post-treatment measure of partisanship is arguably less susceptible to change as a result of the manipulation. Instead of (or in addition to) selecting only partisan participants, it would be beneficial to rerun this study with a larger sample size. The modest sample size in the present study provided a power of 80% for a one-tailed t-test for the main analyses and allowed the detection of an effect size greater than or equal to 0.32, our smallest effect size of interest. Although this is arguably reasonably well-powered for the main analyses conducted and reported here, it limits the possibility of any more fine-grained evaluation of the data and perhaps limited the ability to detect a significant beneficial effect of assessing the micro-level bias on subsequent macro-level bias assessment.

Another limitation of the present study is the fact that the text was available for reference in the micro- but not macro-level condition. This strategy was chosen to emphasize the distinction between the careful, detail-oriented assessment of bias in the micro-level condition and the cursory, overall impression of bias in the macro-level condition, which might best reflect the way news consumers typically assess bias. Moreover, micro-level bias analysis—as operationalized here—might have been very difficult if not impossible to attempt from memory, after only one reading. However, future studies should add a condition in which people are asked about macro-level bias with the article in hand, to see if the option to refer back to the text will have any impact on our overall impression of bias. Conversely, it would be valuable to explore if careful consideration of the article *without* the ability to check the text would affect the results.

Future studies would also benefit from using a number of different articles, of varying length and breadth of news coverage. These articles should also be pre-screened for objectivity by an independent panel rather than relying on the author’s opinion about what is and is not objective. This would allow the exploration and comparison of the specific types of micro-level bias, to see if people are generally more likely to rate certain elements of an article (such as the perceived omission of relevant facts or the accompanying image) as containing more bias than other textual elements. If we could identify the specific factors of a balanced media communication which are most susceptible to being misinterpreted as having a counter-attitudinal bias, perhaps journalists can more carefully attend to these factors when reporting on the news and enhance the probability that an article will be viewed objectively, even by a highly ego-involved reader.

Finally, it would be valuable to investigate how several moderating factors change the relationship between careful and hasty bias evaluation, such as the perceived source and audience of the text, the incongruity of one’s opinion on a given controversial issue with the perceived opinions of others, and, various other individual differences such as intelligence, news consumption habits, political knowledge, and various other measures of cognitive ability (e.g., the Cognitive Reflection Test [28] and Need for Cognition [29]).

Despite the limitations, the present research is an important step in understanding how different levels of deliberation and analysis may impact the perception of bias in the media. The results support the hypothesis that the misperception of bias in the media is largely the result of shallow information processing based on a hasty rather than considered evaluation, and that, once such bias is perceived and reported, it may be very hard to convince readers of the article’s objectivity, even when asking them to pinpoint where exactly the bias might be hiding.

The present research assumes that the micro- (rather than macro-) level approach to the assessment of bias prompts the more accurate judgment. Is it possible, however, that a quick, gestalt analysis of an article may allow the reader to *accurately* identify bias that is not captured by considering the article’s individual features? Although some things, like faces [30] and words [31], are identified holistically (rather than in a constituent manner), it is unlikely that the identification of bias in a news article works like this. In fact, it is hard to imagine how *any* article could be correctly identified as biased in an overall fashion but then not be biased according to the corresponding micro-level measures, which capture everything from the omission of historical context to a skewed distribution of attention devoted to each side.

Future studies can consider additional or alternative explanations for the observed results. A surprising recent finding is that people who spread misinformation online actually care about the accuracy of the news they consume, but simply attend to other dimensions—such as political concordance with their views—when making decisions about the news content [32]. However, priming them to reflect on whether or not the content is true actually lowered their willingness to share false news. In line with these findings, it is perhaps possible that our micro-level bias assessment exercise is priming an “accuracy mindset” while the macro-level assessment is encouraging the more default approach to news assessment through a partisan lens.

The present research provides initial evidence that attentive reading and detailed analysis of a news text may protect against false accusation of bias. But future studies should further investigate this claim with a more partisan (and larger) sample. Of course, as is their nature, people will always continue to see even objective information through the lens of their preconceived notions, but, in our deeply polarized and media-dependent world, it is critical to understand how these errors of reasoning occur and, more importantly, how they might be counteracted.

## Supporting information

S1 File Appendix: Article & main survey questions. (DOCX)

S2 File Raw data (in SPSS). (SAV)
