# Supplementary material for: (Mis)perception of bias in print media: How depth of content evaluation affects the perception of hostile bias in an objective news report
Source: PLoS One. 2021 May 26;16(5):e0251355. doi: 10.1371/journal.pone.0251355 (PMC8153466; doi:10.1371/journal.pone.0251355)
Supplement: S1 File — (DOCX) [file pone.0251355.s001.docx]

# **Appendix: Article Link & Main Survey Questions**

**Original Article Link:**

<https://www.nytimes.com/2015/12/24/world/middleeast/israel-jerusalem-palestinian-stabbing.html>

**Main Survey Questions:**

**[article available to participant]**

[Micro1] The article used poorly chosen language, such as misleading or emotionally charged words that are inappropriate.

[Micro2] The article reported some stretched, exaggerated or incorrect facts about the event.

[Micro3] The article left out some relevant information about the topic which probably should have been included.

[Micro4] The article included some information that was not relevant to this particular event and probably should not have been included.

[Micro5] The article included specific instances of inappropriate opinionated rather than objective reporting.

[Micro6] The attention devoted to the Israeli and Palestinian sides or perspectives was balanced and appropriate.

[Micro7] The article headline was well chosen/descriptive/objective.

[Micro8] The photograph was well chosen/descriptive/objective.

**[article not available to participant]**

[Macro1] Overall, the article treated/presented both sides fairly.

[Macro2] This article is likely to make a reader with a neutral or uncertain position on the Israeli/Palestinian conflict change his or her position and begin supporting either the Israelis or the Palestinians.

[Macro3] Overall, this article was biased — the reporting was not fully objective.

[Macro4] Based on this article, it is likely that the journalist who wrote the story has a

bias in regard to this issue.

[Macro5] Based on this article, it is likely that the newspaper/website which published the

story has an institutional bias in regard to this issue.

**[If bias indicated for any of the questions above, follow-up question asked. Example below:]**

You indicated that you believe the article used poorly chosen language, such as misleading or emotionally charged words that are inappropriate. Do you think this shows that the article is biased against one of the sides (meaning that it is not sufficiently objective)?

1. Yes, the article is biased against Israel/Israelis
2. Yes, the article is biased against the Palestinian territories/Palestinians
3. No, the article is not biased against either side
